# A Dual TLR Agonist Adjuvant Enhances the Immunogenicity and Protective Efficacy of the Tuberculosis Vaccine Antigen ID93

**DOI:** 10.1371/journal.pone.0083884

**Published:** 2014-01-03

**Authors:** Mark T. Orr, Elyse A. Beebe, Thomas E. Hudson, James J. Moon, Christopher B. Fox, Steven G. Reed, Rhea N. Coler

**Affiliations:** 1 Infectious Disease Research Institute, Seattle, Washington, United States of America; 2 Center for Immunology and Inflammatory Diseases and Pulmonary and Critical Care Unit, Massachusetts General Hospital and Harvard Medical School, Charlestown, Massachusetts, United States of America; 3 Department of Global Health, University of Washington, Seattle, Washington, United States of America; University of Delhi, India

## Abstract

With over eight million cases of tuberculosis each year there is a pressing need for the development of new vaccines against *Mycobacterium tuberculosis*. Subunit vaccines consisting of recombinant proteins are an attractive vaccine approach due to their inherent safety compared to attenuated live vaccines and the uniformity of manufacture. Addition of properly formulated TLR agonist-containing adjuvants to recombinant protein vaccines enhances the antigen-specific CD4^+^ T cell response characterized by IFN-γ and TNF, both of which are critical for the control of TB. We have developed a clinical stage vaccine candidate consisting of a recombinant fusion protein ID93 adjuvanted with the TLR4 agonist GLA-SE. Here we examine whether ID93+GLA-SE can be improved by the addition of a second TLR agonist. Addition of CpG containing DNA to ID93+GLA-SE enhanced the magnitude of the multi-functional T_H_1 response against ID93 characterized by co-production of IFN-γ, TNF, and IL-2. Addition of CpG also improved the protective efficacy of ID93+GLA-SE. Finally we demonstrate that this adjuvant synergy between GLA and CpG is independent of TRIF signaling, whereas TRIF is necessary for the adjuvant activity of GLA-SE in the absence of CpG.

## Introduction

Approximately two billion people are infected with *Mycobacterium tuberculosis* (*M.tb.*) the causative agent of tuberculosis (TB). In 2011 there were over 8 million cases of active TB leading to 1.5 million deaths worldwide. The only vaccine against TB, BCG limits childhood disease, but is variably effective against pulmonary TB in adults, with efficacy estimates ranging from 0 to 80% [1]. Thus there is a pressing need for new vaccine candidates against this devastating disease. In the last decade there has been considerable effort to develop new candidate vaccines against TB, with several entering clinical testing [2].

*M.tb.* is primarily controlled by T_H_1 cells producing IFN-γ and TNF that activate infected macrophages, as well as IL-2 which sustains the T cell response [3]. This has led to the hypothesis that the magnitude of the multifunctional T_H_1 cells (i.e. cells producing IFN-γ, TNF, and IL-2) is important for an effective vaccine response against *M.tb*. [4]. Thus enhancing the magnitude of the T_H_1 response against candidate vaccine antigens is critical for developing an effective vaccine against TB. We have developed a clinical candidate antigen, ID93, which is a fusion of four *M.tb*. proteins: Rv1813, Rv2608, Rv3619, and Rv3620. When paired with the adjuvant GLA-SE, prophylactic immunization with ID93+GLA-SE limits *M.tb.* in mice and guinea pigs [5]. Therapeutic vaccination with ID93+GLA-SE is an effective adjunct to chemotherapy in *M.tb.* infected mice and non-human primates [6]. The adjuvant GLA-SE contains the synthetic TLR4 agonist GLA formulated in a stable oil-in-water nano-emulsion (SE) [7], [8]. In the absence of GLA, ID93 formulated in SE elicits a weak, non-protective T_H_2 response in mice. Therefore inclusion of a TLR4 agonist is essential for the efficacy of this vaccine [9]. Additionally the nature of the vaccine formulation can have profound effects on both the immunogenicity and efficacy of ID93+GLA vaccines[10]. ID93+GLA-SE is currently undergoing Phase I clinical testing [11].

Although TLR4 agonists such as GLA and monophosporyl lipid (MPLA) are the most clinically advanced TLR agonist adjuvants for vaccines, with MPLA included in several licensed vaccines, other TLR agonists are also being developed as vaccine adjuvants [12]–[15]. Additionally there is substantial interest in combining TLR agonists as vaccine adjuvants to enhance immunogenicity and efficacy. *In vitro* and *in vivo* studies have found that certain combinations of TLR agonists can cooperate to enhance cytokine and chemokine production by professional antigen presenting cells such as dendritic cells and macrophages, whereas other combinations of agonists can impair vaccine efficacy [16]–[18]. Several groups have found that combining two or three TLR agonists can enhance T cell and antibody responses against experimental vaccines [17], [19]–[21]. In our own work we have shown that combining MPLA or GLA with a TLR9 agonist CpG enhances the efficacy of a candidate therapeutic vaccine against *Leishmaniasis* [22].

The different TLRs signal either through MyD88 (TLR2, TLR5, TLR7, TLR8 and TLR9), TRIF (TLR3) or both signaling pathways (TLR4) [23]. Effective enhancement of adjuvant activity with multiple TLR agonists is most often seen with combinations of agonists that activate the TRIF pathway with agonists that activate the MyD88 pathway. This has led to the hypothesis that MyD88 and TRIF cooperation is necessary for enhanced adjuvant activity with a combination of TLR agonists [16], [17], [24]. We present evidence that combining TLR4 and TLR9 agonists enhances the T_H_1 response against ID93 and leads to increased protection against aerosolized *M.tb.* challenge. Further we explore the contribution of TRIF to this enhanced immunogenicity.

## Materials and Methods

### Ethics statement

The study was conducted under protocol number 2011/5 approved by the Infectious Disease Research Institute Institutional Animal Care and Use Committee.

### Animals and immunizations

6–8 week old female C57BL/6 mice were purchased from Charles River and The Jackson Laboratory and maintained in Specific Pathogen Free conditions. TRIF^−/−^ (also known as *Ticam1^−/−^*) breeder mice on the C57BL/6 background were purchased from The Jackson Laboratory and bred in-house. After infection animals were maintained in ABSL3 containment. Mice were immunized three times three weeks apart by intramuscular injection. Each immunization contained 0.5 µg of ID93 recombinant protein [5] with 5 µg of GLA (Avanti Polar Lipids), 8 µg of CpG1826 (Oligos, Etc.), or both. Adjuvants were formulated in-house in IDRI's stable emulsion (SE), as described previously [8]. For BCG immunization 5×10^4^ CFU (Pasteur strain, Sanofi Pasteur) were injected intradermally once at the time of the first subunit immunization.

### Intracellular cytokine staining

One week after the final immunization splenocytes were isolated from three to five animals per group. Red blood cells were lysed using Red Blood Cell Lysis Buffer (eBioscience) and resuspended in RPMI 1640 and 10% FBS. Cells were plated at 2×10^6^ cells/well in 96-well plates and were stimulated for 1 hour with media or ID93 (10 µg/mL) at 37°C. GolgiPlug (BD Biosciences) was added and the cells were incubated for an additional 7 hours at 37°C. Cells were washed and surface stained with fluorochrome labeled antibodies to CD4 (clone GK1.5), CD8 (clone 53–6. 7), and CD44 (clone IM7) (BioLegend and eBioscience) in the presence of 20% normal mouse serum for 20 minutes at 4°C. Cells were washed and permeabilized with Cytofix/Cytoperm (BD Biosciences) for 20 minutes at room temperature. Cells were washed twice with Perm/Wash (BD Biosciences) and stained intracellularly with fluorochrome labeled antibodies to IFN-γ (clone XMG-1.2), IL-2 (JES6-5H4), TNF (MP6-XT22) (BioLegend and eBioscience), IL-5 (clone TRFK5), and IL-17 (clone TC11-18H10.1) for 20 minutes at room temperature. Cells were washed and resuspended in PBS. Up to 10^6^ events were collected on a four laser LSRII Fortessa flow cytometer (BD Biosciences). Data were analyzed with FlowJo. Cells were gated as singlets > lymphocytes > CD4^+^ CD8^−^ > CD44^+^ > cytokine positive. Analysis and presentation of distributions was performed using SPICE version 5.2, downloaded from <http://exon.niaid.nih.gov/spice.

### MHC class II tetramer production and staining

ID93-specific I-A^b^ tetramers with the immunodominant epitope from Rv3619 (VIYEQANAHGQ) and Rv2608 (AVLPPEVNSA) were produced using methods previously described [25], [26]. One week after the final immunization splenocytes were isolated as described above. Cells were then stained for one hour at room temperature with 10 µM tetramer. Cells were washed and stained for surface CD4, CD8, and CD44. Up to 10^6^ events were collected on a four laser LSRFortessa flow cytometer (BD Biosciences). Cells were gated as singlets > lymphocytes > CD8^−^ CD4^+^ > CD44^+^ > tetramer^+^.

### Mtb aerosol challenge and enumeration

Four to twelve weeks after the last immunization, mice (n = 7/group) were aerogenically infected with *M. tuberculosis* H37Rv (ATCC No. 35718; American Type Culture Collection) using a GlasCol aerosol generator calibrated to deliver 50–100 bacteria into the lungs. To confirm the amount of bacteria delivered an additional three unimmunized animals per infection were euthanized one day later and bacterial burden in the lungs were enumerated. Protection was determined three to four weeks after challenge by harvesting the lungs from the infected mice, homogenizing the tissue in 0.1% PBS–Tween 80, and plating 5-fold serial dilutions on7H10 agar plates (Molecular Toxicology) for bacterial growth. Bacterial colonies were counted after incubation at 37°C with 5% CO_2_ for 14–21 days.

### Histology

Formalin-fixed lung lobes were embedded in paraffin, sectioned and stained with hematoxylin and eosin as a purchased service by the Benaroya Research Institute Histology Core (Seattle, WA). Images were obtained at 10× magnification using a Nikon DS Camera Control Unit DS-L2 on a Nikon Eclipse E400 compound microscope.

### Cytokine production by macrophages

Single cell suspensions of mouse splenocytes were stimulated with media, GLA (1 µg/mL), CpG (10 µg/mL), or both in the presences of Brefeldin A for eight hours using a method adopted from Hajjar *et al.* [27]. Cells were washed with PBS and treated with anti-CD16/CD32 blocking antibody (clone 93). Cells were washed and surface stained with fluorochrome labeled antibodies to CD3 (clone 17A2), CD19 (clone 1D3), CD11b (clone m1/70), and Ly6G (clone 1A8) (BioLegend and eBioscience) for 20 minutes at 4°C. Cells were washed and permeabilized with Cytofix/Cytoperm (BD Biosciences) for 20 minutes at room temperature. Cells were washed twice with Perm/Wash (BD Biosciences) and stained intracellularly with fluorochrome labeled antibodies to IL-12p40 (clone C17.8), TNF, and IL-6 (clone MP5-20F3) (BioLegend and eBioscience) for 20 minutes at room temperature. Cells were washed and resuspended in PBS. Up to 10^6^ events were collected on a four laser LSRII Fortessa flow cytometer (BD Biosciences). Data were analyzed with FlowJo. Cells were gated as singlets > CD3^−^CD19^−^ > not neutrophils (Ly6G^hi^ CD11b^hi^) > macrophages (CD11b^+^) > cytokine positive.

### Statistical methods

Bacterial burdens were normalized by log_10_ transformation. Statistical significance of differences in bacterial burden and cytokine production were determined by analysis of variance using the Bonferroni correction for multiple comparisons using Prism 5 (GraphPad Software).

## Results

### Enhanced T_H_1 responses to ID93 with the combination adjuvant GLA and CpG

**Figure 1:**
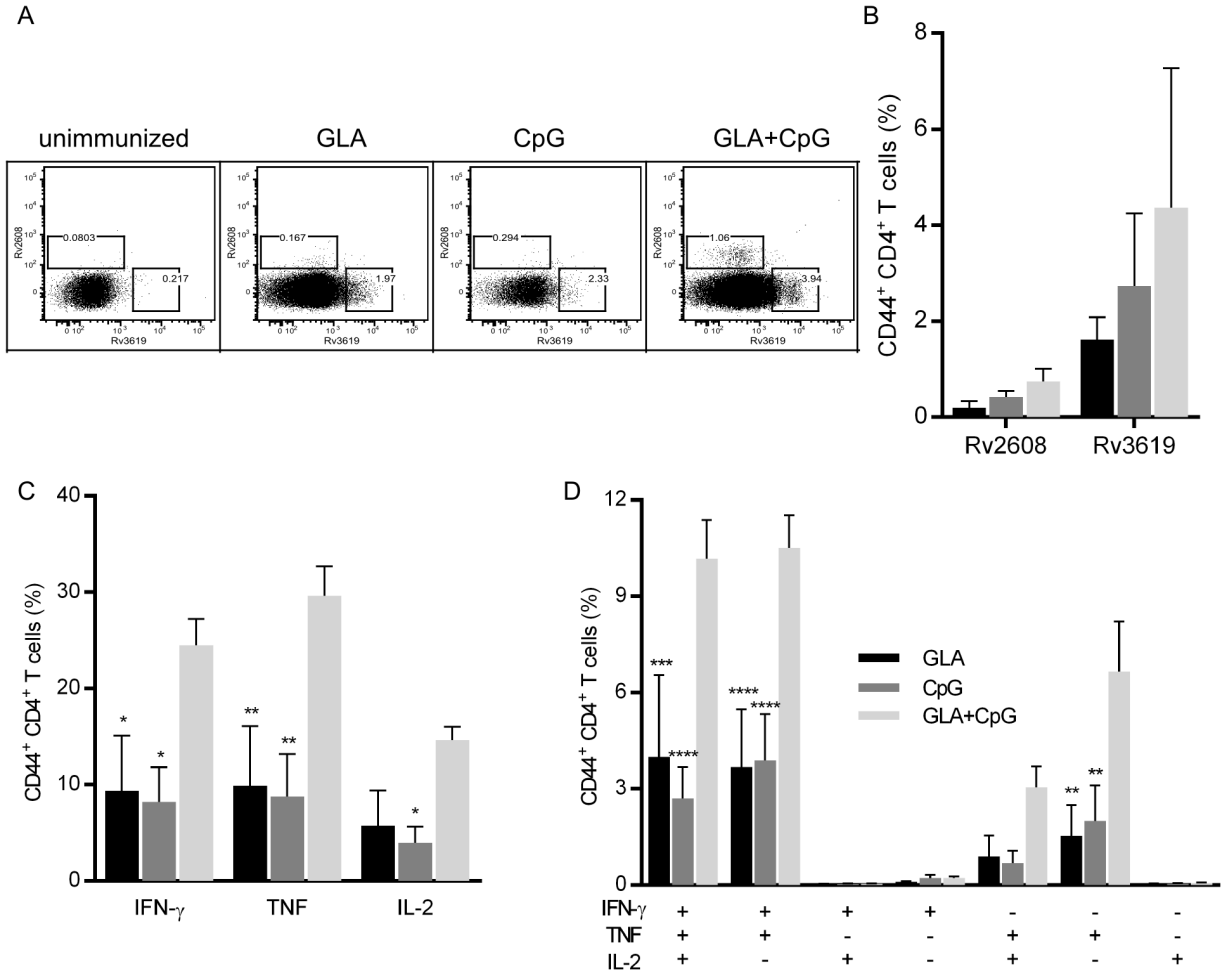
GLA and CpG combine to enhance T_H_1 responses to ID93. Mice were immunized with ID93 adjuvanted with GLA, CpG, or GLA+CpG. (A and B) One week after the final immunization ID93-specific CD4^+^ T cells were identified by staining with I-A^b^ tetramers presenting dominant epitopes from Rv2608 and Rv3619. Cells are gated as singlet, CD4^+^ CD44^+^. Splenic ID93-specific T_H_1 CD4^+^ T cells from immunized mice were identified by cytokine production following *ex-vivo* restimulation with ID93 and analyzed for (C) total cytokine response or (D) poly-functional responses. See Figure S1 for representative cytokine staining. Data are representative of four experiments with similar results with 3–5 animals per group. ^*^,^**^,^***^, and ^****^ indicate *P*<0.05, 0.01, 0.001, and 0.0001 respectively, relative to GLA+CpG as determined by ANOVA using the Bonferroni correction for multiple comparisons.

To determine whether a combined CpG and GLA adjuvant would enhance the CD4^+^ T cell response to ID93 we immunized C57BL/6 mice with ID93 adjuvanted with GLA, CpG, or both. All vaccines were formulated as stable nanodroplet emulsions (SE) [8]. One week after the third immunization we determined the frequency of CD4^+^ T cells specific for two immunodominant epitopes of ID93 by MHC class II tetramer staining. ID93+CpG-SE elicited greater frequencies of ID93-specific CD4^+^ T cells compared to ID93+GLA-SE (Figure 1A and B). Combining the two TLR agonists further enhanced the frequency of ID93-specific CD4^+^ T cells. When splenocytes were stimulated with ID93 and assessed for cytokine production we found that ID93+GLA-SE and ID93+CpG-SE elicited similar frequencies of CD4^+^ T cells producing IFN-γ, TNF, or IL-2 (Figure 1C). We did not detect appreciable levels of IL-17 or IL-5 producing cells from these immunizations (data not shown). Combining GLA and CpG more than doubled the frequency of cytokine producing CD4^+^ T cells (Figure 1C). To determine the extent of multi-functionality of these ID93 specific cells we analyzed the frequency of cells making all combinations of IFN-γ, TNF, and IL-2. The overall distribution of cells was similar across all three immunization regimens, with ID93+CpG/GLA-SE eliciting more cells in all categories (Figure 1D). The vast majority of ID93-specific CD4^+^ T cells produced both IFN-γ and TNF with approximately half of these cells also producing IL-2. Taken together these data demonstrate that the combination adjuvant CpG/GLA-SE augments production of multi-functional T_H_1 cells to a greater extent than either GLA-SE or CpG-SE.

### GLA and CpG cooperate to enhance the protective efficacy of ID93

**Figure 2:**
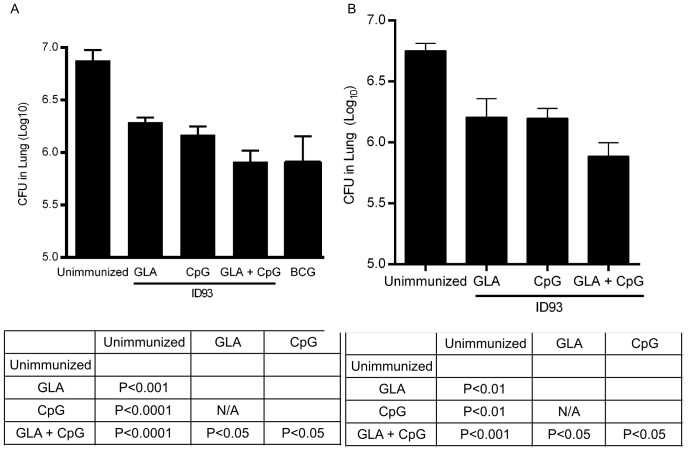
GLA and CpG combine to enhance the protective efficacy of ID93. Mice were immunized and challenged with a low dose of aerosolized *M.tb.* (A) four or (B) twelve weeks later. *M.tb.* burdens in the lungs were determined four weeks after infection. Statistically significant differences between vaccinated groups were determined by ANOVA using the Bonferroni correction for multiple comparisons. Data are representative of three experiments with similar results with seven animals per group.

Although IFN-γ and TNF are critical for control of *M.tb.*, there are conflicting reports regarding the correlation between the magnitude of IFN-γ and TNF producing T cells elicited by vaccination and the degree of protective efficacy [3], [28]–[30]. To determine whether the enhanced T_H_1 response generated by ID93+CpG/GLA-SE resulted in more substantial protection against *M.tb.* we challenged immunized mice with a low dose of aerosolized *M.tb.* one month after the final immunization. ID93+GLA-SE and ID93+CpG-SE immunization both conferred substantial protection against *M.tb.* challenge (P<0.001 and P<0.0001 relative to unimmunized, respectively) (Figure 2A). Combining the two immunostimulants into the same adjuvant formulation further reduced the *M.tb.* burden in the lungs of infected mice to a level induced by BCG immunization (Figure 2A). To determine whether this enhanced protective response was long lasting we challenged a second cohort of immunized mice three months after the final immunization. Again we found that ID93+GLA-SE and ID93+CpG-SE both limited the *M.tb.* burden in the lungs (Figure 2B). The combination adjuvant further reduced the bacterial burden at this late time point, indicating that this superior protective efficacy was durable and significant.

We also assessed cellular infiltration and lung pathology in the animals challenged four weeks after immunization. In unimmunized animals there was a significant reduction in free airway space and substantial numbers of loosely aggregated granulomatous structures (Figure S2). Immunization with ID93+GLA-SE or ID93+CpG-SE resulted in greater free airway space, and fewer areas of consolidated granulomas, however the granulomas that developed were more structured than those seen in the unimmunized animals. Immunization with ID93+CpG/GLA-SE extended this protective benefit, with little sign of consolidated areas in the lungs (Figure S2). Overall these data indicate that the enhanced T_H_1 response elicited with the combined CpG/GLA-SE adjuvant with ID93 correlated with enhanced protection both in terms of reduced bacterial burden and limited lung pathology.

### TRIF is not necessary for enhanced immunogenicity with the combined GLA and CpG adjuvant

**Figure 3:**
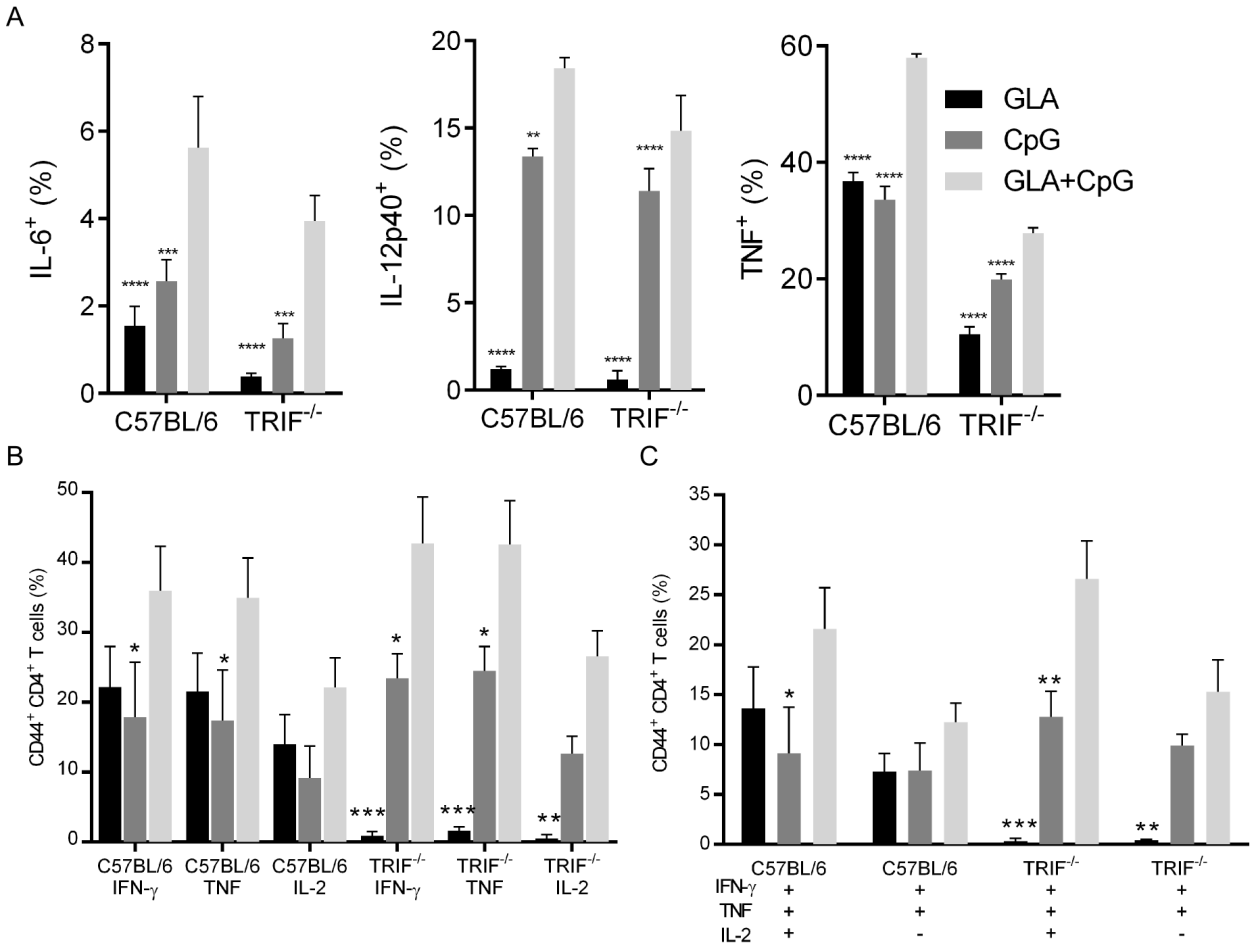
Enhanced T_H_1 responses to GLA and CpG are independent of TRIF signaling. (A) C57BL/6 and TRIF^−/−^ splenocytes were stimulated *ex vivo* with GLA, CpG or both and analyzed for IL-6, IL-12p40, and TNF production by macrophages. (B and C) C57BL/6 and TRIF^−/−^ mice were immunized with ID93 adjuvanted with GLA, CpG or GLA+CpG. Splenic ID93-specific T_H_1 CD^+^4 T cells from immunized mice were identified by cytokine production following ex-vivo restimulation with ID93. Data are representative of two experiments with similar results with 3–5 animals per group. ^*^,^**^,^***^, and ^****^ indicate *P*<0.05, 0.01, 0.001, and 0.0001 respectively, relative to GLA+CpG as determined by ANOVA using the Bonferroni correction for multiple comparisons.

Both MyD88 and TRIF are necessary for the adjuvant activity of GLA-SE [31]. To determine whether TRIF signaling is necessary for GLA to augment the adjuvant activity of CpG-SE we compared the cytokine production by C57BL/6 and TRIF-deficient splenic macrophages stimulated with GLA, CpG, or both. In the absence of TRIF, GLA induction of IL-6, IL-12p40 and TNF was substantially impaired, whereas there was little impact on cytokine induction by CpG in the absence of TRIF (Figure 3A). The combination of GLA and CpG enhanced production of these cytokines in C57BL/6 macrophages and surprisingly this additive effect was also present in TRIF-deficient macrophages, indicating that TRIF may not be necessary for enhanced adjuvant activity of the GLA and CpG combination.

To directly evaluate the contribution of TRIF signaling to the enhanced adjuvant activity of GLA and CpG we immunized B6 and TRIF-deficient mice with ID93 adjuvanted with GLA-SE, CpG-SE, or CpG/GLA-SE. As we saw previously (Figure 1) the combination of GLA and CpG enhanced the frequency of CD4^+^ T cells producing IFN-γ, TNF, and IL-2 upon restimulation with ID93 (Figure 3B and C). In the absence of TRIF, ID93+GLA-SE did not elicit ID93-specific T_H_1 cells. In TRIF-deficient mice the magnitude of the T_H_1 response elicited by the combination GLA and CpG adjuvant was substantially greater than that elicited by CpG alone (Figure 3B and C). Thus GLA and CpG cooperate to enhance the magnitude of the T_H_1 response to immunization in a TRIF-independent fashion.

## Discussion

TB remains one of the most clinically important infectious diseases globally. By examining the breadth of T cell responses to *M.tb.* proteins found in infected patients and evaluating the potential for protection when delivered as a prophylactic vaccine we have identified a set of candidate antigens for inclusion in new subunit vaccine against TB. These antigens were initially identified as protective in animal models when they were paired with a CpG containing adjuvant [32]. We have found that these proteins are also protective when delivered with the TLR4 agonist adjuvant GLA-SE [5]. We now demonstrate that by combining CpG and GLA in the same adjuvant formulation we can further enhance the immunogenicity and protective efficacy of the ID93 vaccine antigen. Further we find that although GLA signaling via the TRIF pathway downstream of TLR4 is necessary for the adjuvant activity of GLA-SE, TRIF is dispensable for the cooperative benefit of combining GLA and CpG.

Infections are first recognized by pattern recognition receptors (PRR) expressed by many cell types including professional APCs such as macrophages and dendritic cells, which prime T cell responses. PRR including TLR, RIG-I like receptors (RLR), C-type lectin receptors (CLR) and NOD-like receptors (NLR) recognize a diverse array of pathogen associated molecular patterns (PAMP). PRR engagement leads to professional APC maturation, increased antigen presentation and secretion of cytokines and chemokines that direct the magnitude and quality of the adaptive immune response [13]. Synthetic and naturally derived PRR agonists have been widely used to mimic infection and shape the immune response to recombinant proteins that would otherwise be poorly immunogenic or tolerogenic [33]. Many infections are detected by multiple PRRs, allowing the immune system to tailor the innate and adaptive immune response to the particular pathogen [34]. For example, *M.tb.* is recognized by TLR2 (MDP), TLR9 (CpG DNA), Mincle (trehalose dimycolate) and NOD2 (MDP) [35].

Many studies have shown that triggering multiple PRR *in vitro* can result in additive, synergistic, or antagonist effects on APC maturation and secretion of cytokines including IL-12, IL-6, and TNF [16], [23], [24], [36]–[41]. This is true both of triggering multiple receptors in the same class, such as multiple TLR agonists, as well as across classes, such as TLR agonists combined with CLR or NLR agonists [42], [43]. Many live and inactivated vaccines including BCG, Typhim, Influvac, and YF-17D engage multiple PRR, which contributes to their immunogenicity and protective efficacy [34], [44]. Seminal work by Napolitani *et al.* demonstrated that conditioning APCs with multiple TLR agonists enhanced their ability to prime T_H_1 responses *in vitro* [16]. Enhanced activity may arise from activation of additional APC types based on receptor expression patterns, augmentation of APC cytokine secretion, or synergistic signaling events in APCs downstream of the different receptors [23]. For example, TLR4 signals through both MyD88 and TRIF, whereas TLR7, 8 and 9 only signal through MyD88. Based on the results of pairwise TLR agonist combinations it has been proposed that signaling through both MyD88 and TRIF were necessary for increased cytokine production and T cell priming [16], [18]. Indeed in the absence of either TRIF or MyD88, synergistic production of cytokines by dendritic cells stimulated with polyI:C and either MALP-2 or CpG was abolished [17]. MyD88 was necessary for the increased CD8^+^ T cell response seen with peptide immunization adjuvanted with MALP-2 and polyI:C [17]. *In vitro* activation of dendritic cells with combinations of TLR3 and TLR9, TLR4 and TLR7, or TLR2 and TLR3 agonists enhanced their ability to induce CD8^+^ T cell responses when transferred in vivo [17], [38], [40]. Despite the plethora of data showing the efficacy of single PRR agonists as vaccine adjuvants, there is surprisingly little data assessing the effects of combining PRR agonists for *in vivo* immunization. We have shown that combining TLR4 and TLR9 agonists enhanced the efficacy of a therapeutic vaccine against Leishmaniasis [22]. Others have found that combining a TLR3 agonist with either a TLR2 or TLR9 agonist, or combining three agonists together can enhance vaccines against *M.tb.* and HIV [17], [19].

We find that the combination of GLA and CpG enhances the production of IL-12 by macrophages *ex vivo*. This correlates well with the increased magnitude of the T_H_1 response to vaccination with both adjuvants. In turn this enhanced CD4^+^ T cell response correlated with improved protection against aerosolized *M.tb.* both in terms of a reduction in bacterial burden and decreased lung pathology. Surprisingly this adjuvant synergy was evident in the absence of TRIF, even though TRIF was necessary for the adjuvant activity of GLA-SE in the absence of CpG. This suggests that although GLA driven MyD88 signaling is not sufficient to enhance T_H_1 production in the absence of TRIF, it is sufficient to augment the MyD88 signaling outcomes driven by CpG triggering of TLR9. Although the exact nature of this synergism in the absence of TRIF is uncertain, it is reasonable to hypothesize that it is dependent on enhanced IL-12 production, which we find to be TRIF-independent when splenic macrophages are stimulated with GLA and CpG. This latter finding expands the possible combinations of TLR agonists that may be beneficial for enhanced adjuvant activity.

We were surprised that the GLA and CpG synergized to enhance IL-12 production by splenic macrophages in a TRIF-independent fashion. A previous study found that TRIF was necessary for enhanced IL-12 production by bone marrow derived dendritic cells stimulated with combinations of LPS and CpG [24]. The difference in our findings and this study may stem from how the particular cell type and preparation or possible differences between LPS and GLA activity, although both signal via TLR4. The discordance between our findings that TRIF is dispensable for adjuvant synergy and previous findings that inclusion of a TRIF-dependent agonist is necessary for adjuvant synergy may be due to differences in model systems. Specifically we have examined the magnitude of the polyclonal T_H_1 response as the measure of adjuvant activity following repeated *in vivo* immunization, whereas previous studies focused on *in vitro* cytokine production by stimulated APCs and/or the ability of APCs stimulated with combinations of TLR agonists *in vitro* to prime T cell responses *in vivo* [16], [18], [38], [40]. Additionally the TRIF-dependence for the synergistic activity of multiple TLR ligands was inferred based on the combinations of stimulants that did or did not enhance activity, rather than testing for adjuvant cooperation in TRIF-deficient models as we have done here.

In summary combining two distinct TLR agonists into an adjuvanted subunit vaccine doubled the magnitude of the T_H_1 response and enhanced the protective efficacy. Until an effective vaccine against TB is developed and tested clinically in efficacy studies it will be impossible to validate correlates of protection. Our current results provide a system to test whether the magnitude of antigen specific CD4^+^ T cells is an important factor for vaccine efficacy in the control of *M.tb.* either for prevention of infection or limitation of active disease.

## Supporting Information

Figure S1 **Representative cytokine gating upon ex-vivo restimulation.** Splenocytes from a C57BL/6 mouse immunized with ID93+GLA were either unstimulated (top row) or restimulated with ID93 in the presence of Brefeldin A. Cells were gated as singlets, lymphocytes, CD44^+^ CD4^+^ CD8^−^ T cells.
(TIF)

Figure S2 **ID93 adjuvanted with GLA and CpG limit lung pathology following** ***M.tb*** **. infection.** Mice were immunized and challenged with a low dose of aerosolized *M.tb.* four weeks later. Four weeks after infection lung sections were stained with H&E to evaluate pathology. Data are representative of three experiments with similar results with four mice per group.
(TIF)

## References

[1] Andersen P, Doherty TM (2005) The success and failure of BCG - implications for a novel tuberculosis vaccine. Nat Rev Microbiol 3: 656–662. 16012514 10.1038/nrmicro1211

[2] Rowland R, McShane H (2011) Tuberculosis vaccines in clinical trials. Expert Rev Vaccines 10: 645–658. 21604985 10.1586/erv.11.28 PMC3409871

[3] Cooper AM (2009) Cell-mediated immune responses in tuberculosis. Annu Rev Immunol 27: 393–422. 19302046 10.1146/annurev.immunol.021908.132703 PMC4298253

[4] Darrah PA, Patel DT, De Luca PM, Lindsay RW, Davey DF, et al (2007) Multifunctional TH1 cells define a correlate of vaccine-mediated protection against Leishmania major. Nat Med 13: 843–850. 17558415 10.1038/nm1592

[5] Bertholet S, Ireton GC, Ordway DJ, Windish HP, Pine SO, et al (2010) A defined tuberculosis vaccine candidate boosts BCG and protects against multidrug-resistant Mycobacterium tuberculosis. Sci Transl Med 2: 53ra74. 10.1126/scitranslmed.3001094 PMC3110937 20944089

[6] Coler RN, Bertholet S, Pine SO, Orr MT, Reese V, et al. (2012) Therapeutic Immunization against Mycobacterium tuberculosis Is an Effective Adjunct to Antibiotic Treatment. J Infect Dis. 10.1093/infdis/jis425 PMC3693588 22891286

[7] Coler RN, Baldwin SL, Shaverdian N, Bertholet S, Reed SJ, et al (2010) A synthetic adjuvant to enhance and expand immune responses to influenza vaccines. PLoS One 5: e13677. 21060869 10.1371/journal.pone.0013677 PMC2965144

[8] Coler RN, Bertholet S, Moutaftsi M, Guderian JA, Windish HP, et al (2011) Development and characterization of synthetic glucopyranosyl lipid adjuvant system as a vaccine adjuvant. PLoS One 6: e16333. 21298114 10.1371/journal.pone.0016333 PMC3027669

[9] Baldwin SL, Bertholet S, Reese VA, Ching LK, Reed SG, et al (2012) The importance of adjuvant formulation in the development of a tuberculosis vaccine. J Immunol 188: 2189–2197. 22291184 10.4049/jimmunol.1102696 PMC3288309

[10] Orr MT, Fox CB, Baldwin SL, Sivananthan SJ, Lucas E, et al (2013) Adjuvant formulation structure and composition are critical for the development of an effective vaccine against tuberculosis. J Control Release 172: 190–200. 23933525 10.1016/j.jconrel.2013.07.030 PMC3871206

[11] Meyer J, McShane H (2013) The next 10 years for tuberculosis vaccines: do we have the right plans in place? Expert Rev Vaccines 12: 443–451. 23560924 10.1586/erv.13.19 PMC5425624

[12] Manicassamy S, Pulendran B (2009) Modulation of adaptive immunity with Toll-like receptors. Semin Immunol 21: 185–193. 19502082 10.1016/j.smim.2009.05.005 PMC4125416

[13] Duthie MS, Windish HP, Fox CB, Reed SG (2011) Use of defined TLR ligands as adjuvants within human vaccines. Immunol Rev 239: 178–196. 21198672 10.1111/j.1600-065X.2010.00978.x PMC5872835

[14] Didierlaurent AM, Morel S, Lockman L, Giannini SL, Bisteau M, et al (2009) AS04, an aluminum salt- and TLR4 agonist-based adjuvant system, induces a transient localized innate immune response leading to enhanced adaptive immunity. J Immunol 183: 6186–6197. 19864596 10.4049/jimmunol.0901474

[15] Schwarz TF (2009) Clinical update of the AS04-adjuvanted human papillomavirus-16/18 cervical cancer vaccine, Cervarix. Adv Ther 26: 983–998. 20024678 10.1007/s12325-009-0079-5

[16] Napolitani G, Rinaldi A, Bertoni F, Sallusto F, Lanzavecchia A (2005) Selected Toll-like receptor agonist combinations synergistically trigger a T helper type 1-polarizing program in dendritic cells. Nat Immunol 6: 769–776. 15995707 10.1038/ni1223 PMC3760217

[17] Zhu Q, Egelston C, Vivekanandhan A, Uematsu S, Akira S, et al (2008) Toll-like receptor ligands synergize through distinct dendritic cell pathways to induce T cell responses: implications for vaccines. Proc Natl Acad Sci U S A 105: 16260–16265. 18845682 10.1073/pnas.0805325105 PMC2570973

[18] Bagchi A, Herrup EA, Warren HS, Trigilio J, Shin HS, et al (2007) MyD88-dependent and MyD88-independent pathways in synergy, priming, and tolerance between TLR agonists. J Immunol 178: 1164–1171. 17202381 10.4049/jimmunol.178.2.1164

[19] Zhu Q, Egelston C, Gagnon S, Sui Y, Belyakov IM, et al (2010) Using 3 TLR ligands as a combination adjuvant induces qualitative changes in T cell responses needed for antiviral protection in mice. J Clin Invest 120: 607–616. 20101095 10.1172/JCI39293 PMC2811160

[20] Kasturi SP, Skountzou I, Albrecht RA, Koutsonanos D, Hua T, et al (2011) Programming the magnitude and persistence of antibody responses with innate immunity. Nature 470: 543–547. 21350488 10.1038/nature09737 PMC3057367

[21] Matthews K, Chung NP, Klasse PJ, Moore JP, Sanders RW (2012) Potent induction of antibody-secreting B cells by human dermal-derived CD14+ dendritic cells triggered by dual TLR ligation. J Immunol 189: 5729–5744. 23162132 10.4049/jimmunol.1200601 PMC3951118

[22] Raman VS, Bhatia A, Picone A, Whittle J, Bailor HR, et al (2010) Applying TLR synergy in immunotherapy: implications in cutaneous leishmaniasis. J Immunol 185: 1701–1710. 20601594 10.4049/jimmunol.1000238 PMC3109724

[23] Trinchieri G, Sher A (2007) Cooperation of Toll-like receptor signals in innate immune defence. Nat Rev Immunol 7: 179–190. 17318230 10.1038/nri2038

[24] Krummen M, Balkow S, Shen L, Heinz S, Loquai C, et al (2010) Release of IL-12 by dendritic cells activated by TLR ligation is dependent on MyD88 signaling, whereas TRIF signaling is indispensable for TLR synergy. J Leukoc Biol 88: 189–199. 20360404 10.1189/jlb.0408228

[25] Moon JJ, Chu HH, Hataye J, Pagan AJ, Pepper M, et al (2009) Tracking epitope-specific T cells. Nat Protoc 4: 565–581. 19373228 10.1038/nprot.2009.9 PMC3517879

[26] Moon JJ, Chu HH, Pepper M, McSorley SJ, Jameson SC, et al (2007) Naive CD4(+) T cell frequency varies for different epitopes and predicts repertoire diversity and response magnitude. Immunity 27: 203–213. 17707129 10.1016/j.immuni.2007.07.007 PMC2200089

[27] Hajjar AM, Ernst RK, Fortuno ES 3rd, Brasfield AS, Yam CS, et al (2012) Humanized TLR4/MD-2 mice reveal LPS recognition differentially impacts susceptibility to Yersinia pestis and Salmonella enterica. PLoS Pathog 8: e1002963. 23071439 10.1371/journal.ppat.1002963 PMC3469661

[28] Kagina BM, Abel B, Scriba TJ, Hughes EJ, Keyser A, et al (2010) Specific T cell frequency and cytokine expression profile do not correlate with protection against tuberculosis after bacillus Calmette-Guerin vaccination of newborns. Am J Respir Crit Care Med 182: 1073–1079. 20558627 10.1164/rccm.201003-0334OC PMC2970848

[29] Mittrucker HW, Steinhoff U, Kohler A, Krause M, Lazar D, et al (2007) Poor correlation between BCG vaccination-induced T cell responses and protection against tuberculosis. Proc Natl Acad Sci U S A 104: 12434–12439. 17640915 10.1073/pnas.0703510104 PMC1941486

[30] Derrick SC, Yabe IM, Yang A, Morris SL (2011) Vaccine-induced anti-tuberculosis protective immunity in mice correlates with the magnitude and quality of multifunctional CD4 T cells. Vaccine 29: 2902–2909. 21338678 10.1016/j.vaccine.2011.02.010

[31] Orr MT, Duthie MS, Windish HP, Lucas EA, Guderian JA, et al (2013) MyD88 and TRIF synergistic interaction is required for TH1-cell polarization with a synthetic TLR4 agonist adjuvant. Eur J Immunol 43: 2398–2408. 23716300 10.1002/eji.201243124 PMC3803998

[32] Bertholet S, Ireton GC, Kahn M, Guderian J, Mohamath R, et al (2008) Identification of human T cell antigens for the development of vaccines against Mycobacterium tuberculosis. J Immunol 181: 7948–7957. 19017986 10.4049/jimmunol.181.11.7948 PMC2586986

[33] Olive C (2012) Pattern recognition receptors: sentinels in innate immunity and targets of new vaccine adjuvants. Expert Rev Vaccines 11: 237–256. 22309671 10.1586/erv.11.189

[34] Querec T, Bennouna S, Alkan S, Laouar Y, Gorden K, et al (2006) Yellow fever vaccine YF-17D activates multiple dendritic cell subsets via TLR2, 7, 8, and 9 to stimulate polyvalent immunity. J Exp Med 203: 413–424. 16461338 10.1084/jem.20051720 PMC2118210

[35] Kleinnijenhuis J, Oosting M, Joosten LA, Netea MG, Van Crevel R (2011) Innate immune recognition of Mycobacterium tuberculosis. Clin Dev Immunol 2011: 405310. 21603213 10.1155/2011/405310 PMC3095423

[36] Roelofs MF, Joosten LA, Abdollahi-Roodsaz S, van Lieshout AW, Sprong T, et al (2005) The expression of toll-like receptors 3 and 7 in rheumatoid arthritis synovium is increased and costimulation of toll-like receptors 3, 4, and 7/8 results in synergistic cytokine production by dendritic cells. Arthritis Rheum 52: 2313–2322. 16052591 10.1002/art.21278

[37] Makela SM, Strengell M, Pietila TE, Osterlund P, Julkunen I (2009) Multiple signaling pathways contribute to synergistic TLR ligand-dependent cytokine gene expression in human monocyte-derived macrophages and dendritic cells. J Leukoc Biol 85: 664–672. 19164128 10.1189/jlb.0808503

[38] Warger T, Osterloh P, Rechtsteiner G, Fassbender M, Heib V, et al (2006) Synergistic activation of dendritic cells by combined Toll-like receptor ligation induces superior CTL responses in vivo. Blood 108: 544–550. 16537810 10.1182/blood-2005-10-4015

[39] Mitchell D, Yong M, Schroder W, Black M, Tirrell M, et al (2010) Dual stimulation of MyD88-dependent Toll-like receptors induces synergistically enhanced production of inflammatory cytokines in murine bone marrow-derived dendritic cells. J Infect Dis 202: 318–329. 20524851 10.1086/653499

[40] Bohnenkamp HR, Papazisis KT, Burchell JM, Taylor-Papadimitriou J (2007) Synergism of Toll-like receptor-induced interleukin-12p70 secretion by monocyte-derived dendritic cells is mediated through p38 MAPK and lowers the threshold of T-helper cell type 1 responses. Cell Immunol 247: 72–84. 17927969 10.1016/j.cellimm.2007.07.008

[41] Gautier G, Humbert M, Deauvieau F, Scuiller M, Hiscott J, et al (2005) A type I interferon autocrine-paracrine loop is involved in Toll-like receptor-induced interleukin-12p70 secretion by dendritic cells. J Exp Med 201: 1435–1446. 15851485 10.1084/jem.20041964 PMC2213193

[42] Tada H, Aiba S, Shibata K, Ohteki T, Takada H (2005) Synergistic effect of Nod1 and Nod2 agonists with toll-like receptor agonists on human dendritic cells to generate interleukin-12 and T helper type 1 cells. Infect Immun 73: 7967–7976. 16299289 10.1128/IAI.73.12.7967-7976.2005 PMC1307098

[43] Underhill DM (2007) Collaboration between the innate immune receptors dectin-1, TLRs, and Nods. Immunol Rev 219: 75–87. 17850483 10.1111/j.1600-065X.2007.00548.x

[44] Schreibelt G, Benitez-Ribas D, Schuurhuis D, Lambeck AJ, van Hout-Kuijer M, et al (2010) Commonly used prophylactic vaccines as an alternative for synthetically produced TLR ligands to mature monocyte-derived dendritic cells. Blood 116: 564–574. 20424184 10.1182/blood-2009-11-251884

