# Supplementary figures and images for: A Dual TLR Agonist Adjuvant Enhances the Immunogenicity and Protective Efficacy of the Tuberculosis Vaccine Antigen ID93
Source: PLoS One. 2014 Jan 3;9(1):e83884. doi: 10.1371/journal.pone.0083884 (PMC3880254; doi:10.1371/journal.pone.0083884)

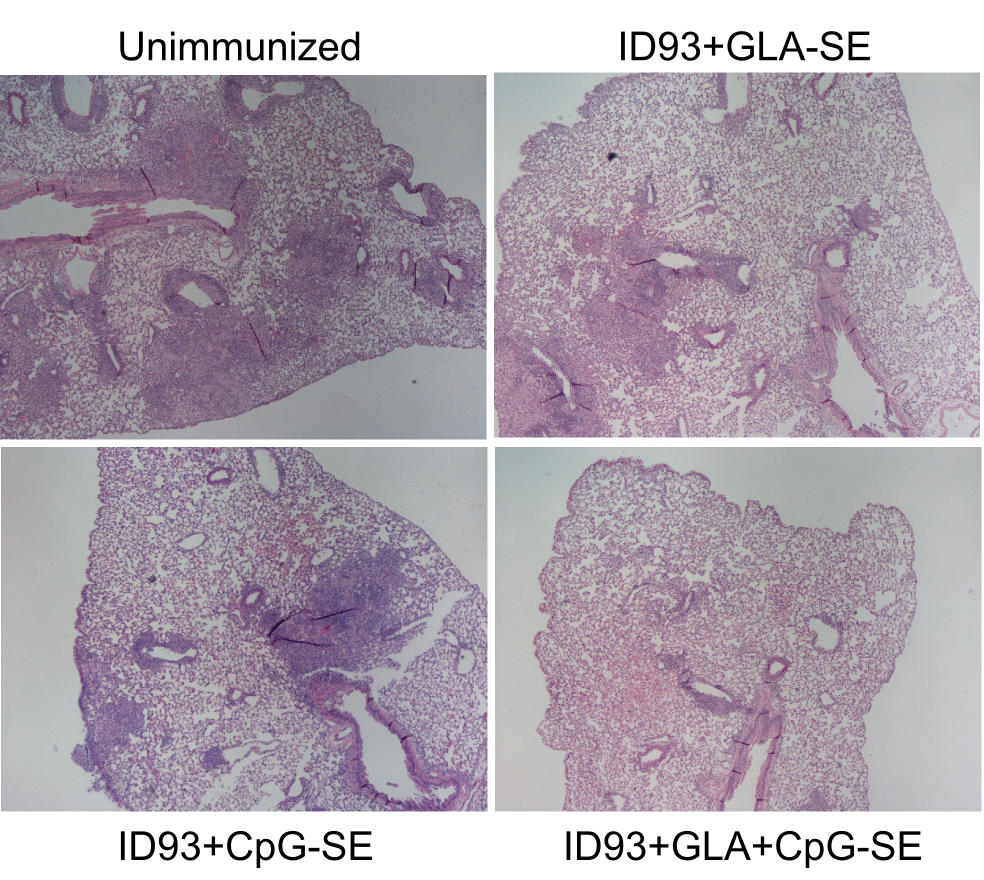

Supplement: Figure S2 — ID93 adjuvanted with GLA and CpG limit lung pathology following M.tb . infection. Mice were immunized and challenged with a low dose of aerosolized M.tb. four weeks later. Four weeks after infection lung sections were stained with H&E to evaluate pathology. Data are representative of three experiments with similar results with four mice per group. (TIF) [file pone.0083884.s002.tif]
